# Abortions and Congenital Malformations in Small Ruminants Associated with Toxic Plant Consumption in the Brazilian Semi-Arid Region

**DOI:** 10.3390/ani15030356

**Published:** 2025-01-26

**Authors:** Valdemar C. Rocha, Givanildo J. Santos Filho, Maria de Fátima de Souza, Edson B. Assis, Misael A. da Silva, Mônica S. Sousa, Eduardo Sérgio S. Sousa, Sara V. D. Simões, Ricardo B. Lucena

**Affiliations:** 1 Department of Veterinary Sciences, Center of Agricultural Sciences, Universidade Federal da Paraiba, Rodovia 079—Km 12, Areia 58397-000, Paraiba, Brazil; valdemar_cavalcante@hotmail.com (V.C.R.); givanildogjfilho@gmail.com (G.J.S.F.); saravdsimoes@gmail.com (S.V.D.S.); 2 Graduate Program in Animal Health and Science, Center for Rural Health and Technology, Universidade Federal de Campina Grande, Avenida Universitária, s/n Bairro Santa Cecília, Patos 58708-110, Paraiba, Brazil; fatima_vet35@hotmail.com; 3 Graduate Program in Animal Science, Center of Agricultural Sciences, Universidade Federal da Paraiba, Rodovia 079—Km 12, Areia 58397-000, Paraiba, Brazil; edson.assis@academico.ufpb.br (E.B.A.J.); monica.shinneider.sousa@academico.ufpb.br (M.S.S.); 4 Undergraduate Program in Animal Science, Center of Agricultural Sciences, Universidade Federal da Paraiba, Rodovia 079—Km 12, Areia 58397-000, Paraiba, Brazil; misael.alves@academico.ufpb.br; 5 Department of Obstetrics and Gynecology, Center for Medical Sciences, Universidade Federal da Paraiba, João Pessoa 58051-900, Paraiba, Brazil; esergiosousa@uol.com.br

**Keywords:** abortions, climate, drought, management, native vegetation, small ruminants, teratogenesis

## Abstract

The Northeast of Brazil is a leading region for small ruminants. However, these herds are primarily located in the semi-arid region and face significant losses due to fetal and perinatal mortality, frequently linked to the consumption of teratogenic toxic plants during pregnancy. This study investigated the clinical and epidemiological aspects related to abortions and malformations in 80 small ruminant farms in the semi-arid region of Brazil. Necropsies were also performed on fetuses and neonates, along with histopathological, molecular, and serological analyses of the herds to differentiate other conditions. Malformations were identified on approximately 42 farms, with arthrogryposis being the most common abnormality. Toxic plants, including *Mimosa tenuiflora* and *Cenostigma pyramidalis*, were recorded on 95.23% of the properties with reported malformations. Additionally, necropsies confirmed the association between the occurrence of these plants and malformations, abortions, and mortality, excluding infectious causes. These results underscore the urgent need for prevention and control strategies to mitigate the impact of toxic plants on small ruminant production in the semi-arid region.

## Simple Summary

Goat and sheep herds in Brazil are primarily concentrated in the semi-arid region of the Northeast, playing a vital role in the local and regional economy. However, these farms face substantial challenges due to the extensive farming system with low technological input and the effects of the local climate, characterized by prolonged drought periods. These factors lead to increased access to and ingestion of toxic plants by the animals, including vegetation that induces teratogenic alterations. Using a questionnaire and in situ observations, we investigated 80 farms in the semi-arid region, encompassing a population of over 6000 small ruminants. We determined that congenital defects occurred in animals raised under semi-intensive and extensive farming systems, which expose pregnant goats and sheep to toxic plants. These herds had access to two proven teratogenic plants, *Mimosa tenuiflora* and *Cenostigma* (*Poincianella*) *pyramidalis*, including during the gestation of goats and ewes. Kids and lambs exhibited severe birth defects such as arthrogryposis, cleft lips and palates, and facial deformities. These findings confirm a high rate of malformations in herds from the semi-arid region, correlated with the effects of toxic plants. This underscores the urgent need for the implementation of preventive measures on farms.

## 1. Introduction

Brazil is one of the leading countries in agricultural and livestock production, including small ruminant farming. In South America, for instance, Brazil accounts for accounting for approximately 44% and 28% of the goat and sheep herds [1], respectively. These herds are primarily concentrated in the Northeast region of the country, which is home to approximately 96% of the goat population and 71.2% of the sheep population. The majority of these animals are raised on farms in the semi-arid region [2].

Small ruminant production in the semi-arid region of Brazil predominantly involves an extensive system, where most producers use family-based and subsistence production systems with low technological input and herds of up to 100 animals. These systems are characterized by traditional management practices and the predominant use of family labor. Animals are generally left to graze freely during the day and are sheltered at night in basic infrastructure, such as corrals and sheds made of wood and clay tiles [3]. During critical periods, such as the dry season or on dairy farms, this system is adapted into a semi-intensive system, which combines pasture grazing with supplemental feeding and occasional confinement [4]. This management approach allows animals to graze on native vegetation while ensuring additional nutritional support when natural forage availability is limited [5].

As a result of the management systems adopted, the small ruminant herds in this Brazilian region face significant challenges due to losses caused by abortions and/or high perinatal mortality attributable to congenital malformations [6,7,8]. Despite the limited data on reproductive losses caused by abortions, infertility, and malformations in Northeast Brazil, studies in the semi-arid region indicate that approximately 10% of neonatal and perinatal deaths in kids and 23% in lambs are caused by malformations, resulting in an estimated annual reduction of 273,120 kids and 259,582 lambs [9].

Toxic plants cause significant economic losses to Brazilian livestock farming [10]. Previous studies have demonstrated that malformations in small ruminants in the Northeast of Brazil are commonly caused by the ingestion of toxic plants during the gestational period of goats and sheep [11,12,13,14]. The plants *Mimosa tenuiflora* and *Cenostigma* (*Poincianella*) *pyramidalis*, which are found in the Brazilian semi-arid region, have been proven to cause teratogenic effects in goat, sheep, and cattle fetuses, as well as in laboratory animals [12,15,16,17,18,19,20,21]. The plant *Aspidosperma pyrifolium* is also considered an important cause of abortion in goats in the region; however, it has not been associated with the occurrence of malformations [22].

*Stryphnodendron fissuratum* is also a plant that induces malformations and abortions [22,23]; however, this plant does not grow in the semi-arid region. Other Brazilian plants can also cause abortions, but they are not associated with the occurrence of malformations [8]. Additional significant causes of reproductive problems in Brazilian herds include infectious agents such as bacteria and protozoa, which should be investigated in the event of abortion outbreaks in herds [24,25,26].

Although previous research has investigated the causes of abortions in Brazilian herds, to the best of the authors’ knowledge, no studies have yet conducted an in-depth epidemiological investigation or long-term follow-up regarding the occurrence of abortions and malformations in small ruminants associated with the consumption of toxic plants. Thus, the present study aimed to investigate the relationship between outbreaks of malformations, abortions, and mortality in small ruminant herds in the semi-arid region and the history of teratogenic plant consumption during gestation. Additionally, this study sought to determine the occurrence of macro- and histopathological lesions in fetuses and perform differential diagnoses to rule out other agents unrelated to toxic plants.

## 2. Materials and Methods

### 2.1. Study Location

The “Cariri Paraibano” microregion is situated on the Borborema Plateau, in the semi-arid area of the State of Paraíba, Brazil, between latitudes 07° and 08°30′ S and longitudes 36° and 37°30′ W. This region is characterized by an average annual temperature of 25 °C and an annual rainfall of approximately 300 mm. The vegetation consists of forests with frequently thorny trees, 4–6 m in height, deciduous in nature, and often accompanied by a layer of soil covered with small deciduous shrubs and annual herbs [27].

### 2.2. Investigation

The study was conducted in two stages: (1) an epidemiological and clinical investigation on farms and (2) the analysis of goat and sheep fetuses and stillbirths through necropsy.

#### 2.2.1. Clinical–Epidemiological Investigation

In the first stage, 80 farms located in the “Cariri Paraibano” region were selected using non-probabilistic sampling, with goat and sheep farming as the sole predetermined inclusion criterion. Preliminary data from the Academic Extension Program (PROBEX) of the Federal University of Paraíba, which provides rural assistance to various municipalities in the semi-arid region, were used for farm selection.

To collect data, animal owners completed a questionnaire covering the following topics: farm identification; purpose of farming (milk or meat production); pasture area in hectares; deforested and native forest area; farming system (intensive, semi-intensive, or extensive); flock size by age group; number of kids born in the past year; sanitary management practices (vaccination, antiparasitic treatment); number of animals with malformations in the past year; types of malformations in young animals; occurrence of abortions and estrus repetitions; use of zootechnical control (herd performance indicators); animal feeding management; mineral supplementation; degree of inbreeding in the herd; and the presence of toxic native plants. After interviewing the farmers, the research team surveyed the grazing areas to identify the presence of teratogenic toxic plants.

#### 2.2.2. Pathological, Molecular, and Serological Investigation

In the second stage of the study, macroscopic, histopathological, and microbiological evaluations were conducted over one year on goat and sheep fetuses, stillbirths, and neonates submitted for necropsy, as well as placentas (in cases of abortions and stillbirths). Animals with congenital malformations that died within five months of age due to complications related to these malformations were included in the study.

During necropsy, all macroscopic abnormalities were recorded, and tissue samples were collected from all organs, including the placenta, when available and preserved in 10% buffered formalin. For histopathological processing, after fixation, the samples were trimmed, routinely processed in alcohol and xylene, embedded in paraffin, sectioned at 5 µm, and stained with hematoxylin and eosin (HE).

Fragments of the placenta, brain, spinal cord, lungs, liver, spleen, kidneys, and cavity fluids were frozen for subsequent specific diagnostic tests to confirm the presence or absence of infectious agents in the tissues. Diagnostic tests were selected based on clinical suspicions, necropsy findings, and histopathological results.

For cases with hemorrhagic lesions and hepatic and renal necrosis suggestive of leptospirosis, frozen tissue fragments were analyzed using polymerase chain reaction (PCR) [28]. Deoxyribonucleic acid (DNA) was extracted using the DNeasy Blood and Tissue Kit (Qiagen, Hilden, Germany), following the manufacturer’s instructions. PCR was performed with the primers LipL32-45F (5′-AAG CAT TAC CGC TGG TG-3′) and LipL32-286R (5′-GAA CTC CCA TTT CAG CGA TT-3′) to amplify the LipL32 gene, which is specific to pathogenic leptospires. The *Leptospira interrogans* serogroup Pomona serovar Kennewicki was used as a positive control, and ultrapure water was used as a negative control. The technique was conducted according to the description by Stoddard et al. (2009) [28].

For the diagnosis of protozoa, genomic DNA was extracted from tissues, and PCR amplification was performed following the methods described in the literature [29].

Additionally, blood was collected in Vacutainer tubes without anticoagulant from 10% of the reproductive-age females with a history of reproductive failure on each farm, followed by centrifugation to obtain serum. The sera were stored in labeled microtubes corresponding to each farm and kept at −20 °C until serological tests were performed based on clinical suspicion, necropsy findings, and results from other tests.

Following histopathological examination of the analyzed fetuses, The sera from the dams of fetuses with leptospirosis were tested using the microscopic agglutination test (MAT) [30]. A panel of 24 pathogenic serogroups: Australis, Bratislava, Autumnalis, Butembo, Castellonis, Bataviae, Canicola, Whitcombi, Cynopteri, Grippotyphosa, Hebdomadis, Copenhageni, Icterohaemorrhagiae, Javanica, Panamá, Pomona, Pyrogenes, Hardjo, Wolffi, Shermani, Tarassovi, Andamana, Patoc, and Sentot. The initial dilution was 1:50, followed by serial twofold dilutions. The highest titer obtained was considered the infecting serogroup.

### 2.3. Statistical Analysis

Statistical analysis was conducted to determine whether there was a correlation between the farming system adopted or the presence of toxic plants on each of the 80 farms and the occurrence of congenital malformations. The number of malformations observed on each farm was used as the dependent variable. Initially, a descriptive analysis was performed to calculate the means and standard deviations of malformations across the different farming systems.

The Shapiro–Wilk test was used to assess the normality of the data, with a significance level of 5%, and confirmed that the data did not follow a normal distribution. Consequently, the Mann–Whitney U test was employed to compare the distributions of malformations between the two farming systems with reported malformations (semi-intensive and extensive). Additionally, Spearman’s correlation coefficient was used to investigate the monotonic relationship between the farming system and the number of malformations.

To examine the relationship between the presence of teratogenic plants on the farms and the occurrence of malformations in the animals, three main variables were used: the presence or absence of each toxic plant (*M. tenuiflora* and *C. pyramidalis*) and the presence of both plants on each farm. Pearson’s correlation analysis was initially performed to determine the strength and significance of the relationship between the occurrence of teratogenic plants and the number of malformations. Subsequently, analysis of variance (ANOVA) and the Kruskal–Wallis test were applied. ANOVA was used to determine significant differences in the number of malformations among groups categorized by the total number of plants, while the Kruskal–Wallis test served as a robust non-parametric alternative to confirm the results.

Two regression models were fitted to quantify the impact of the occurrence of both plants on the number of malformations. A Poisson regression model was applied due to the count nature of the dependent variable. However, the detection of overdispersion (variance greater than the mean) justified the adjustment of a negative binomial regression model, which provided a better fit to the data.

To evaluate the relationship between the adoption of zootechnical control and the occurrence of malformations in small ruminants, a cross-sectional study was conducted on 42 farms. The farms were divided into two groups: those implementing zootechnical control and those not implementing this control. The total number of malformations reported on each farm was recorded as the dependent variable. For the statistical analysis, a point-biserial correlation was used to assess the association between zootechnical control (a binary variable) and the number of malformations (a continuous variable). Additionally, Student’s *t*-test was applied to compare the means between the two groups, accounting for unequal variances.

All statistical analyses were performed using Python software (versions 3.11 and 3.8), with scipy (version 1.11.1), pandas (version 1.5.3), and scipy.stats packages. The significance level adopted for all tests was 5%.

## 3. Results

### 3.1. Farm Characteristics and Occurrence of Congenital Malformations

In the 80 farms studied, a total population of 6909 animals was identified, consisting of 5988 goats and 921 sheep. The largest farm housed a herd of 600 animals, while the smallest managed just 15, with an average of approximately 86 animals per property. The characteristics of these farms are detailed in total numbers and percentages in Table 1.

Regarding the number of animals per farm, the smallest farm had a herd of 21 animals, while the largest had 600 animals. Among the farms, 71.25% (57/80) adopted a semi-intensive farming system, 27.5% (22/80) used an extensive system, and only 1.25% (1/80) employed an intensive farming system. Mineral supplementation was adopted by 70% (56/80) of the farms.

In the studied population of small ruminants, 6.01% (415) of the animals were identified with malformations, comprising 6.28% (376/5988) among goats and 4.23% (39/921) among sheep. Among the animals with malformations, 414 were less than six months old, while only one was an adult. It was found that, in the age group of up to six months, 30.7% (415/1352) of the animals were affected by malformations.

**Figure 1:**
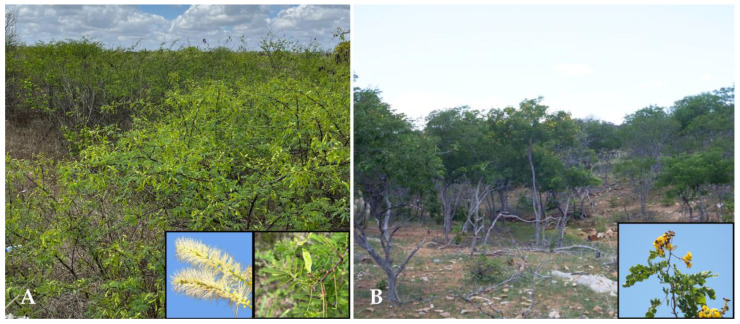
Areas infested by teratogenic toxic plants. (**A**) Farm with a large presence of *Mimosa tenuiflora* showing the plant in its flowering and fruiting stage (inset). (**B**) Farm with a high prevalence of *Cenostigma pyramidalis*, showing the plant in its flowering stage (inset).

Cases of malformations were identified on 52.5% (42/80) of the studied farms. The investigation confirmed that 95.23% (40/42) of these farms had a high prevalence of the teratogenic plants *M. tenuiflora* and *C. pyramidalis* (Figure 1) in grazing areas accessible to goats and sheep during gestation.

**Figure 2:**
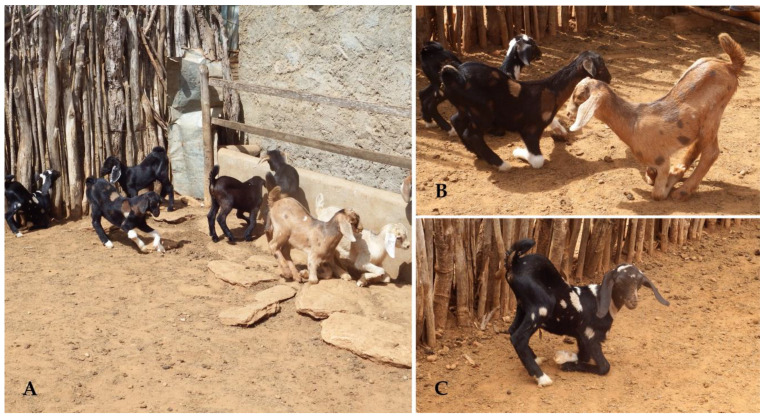
Congenital malformations associated with the consumption of teratogenic plants. Goat kids with arthrogryposis. (**A**) Arthrogryposis observed in a large number of animals on the same farm. (**B**) Goat kids born alive exhibiting arthrogryposis primarily affecting the thoracic limbs. (**C**) Arthrogryposis significantly hinders the animal’s movements.

**Figure 3:**
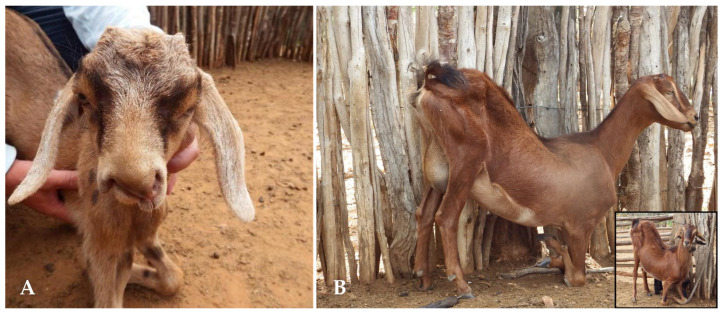
Congenital malformations associated with the consumption of teratogenic plants. (**A**) Young goat kid affected by a nasolabial cleft and arthrogryposis. (**B**) Adult goat affected by arthrogryposis. Goat viewed from the front (inset).

The clinical evaluation of animals affected by malformations revealed a variety of abnormalities, with arthrogryposis and cleft lips (Figure 2 and Figure 3) being the most prevalent, particularly in young animals. These malformations were rarely observed in adult animals (Figure 3B).

Congenital malformations were identified in approximately 6% of the goat herd and 4% of the sheep herd. The different types of congenital malformations observed in the kids and lambs examined on the farms are detailed in Table 2. Clinical evaluation of the remaining animals in the herds, including pregnant females, did not reveal any other abnormalities associated with the consumption of toxic plants.

### 3.2. Fetuses with Malformations

A total of 54 fetuses, stillbirths, or neonates were submitted for necropsy, of which 40 (74%) were goats and 14 (26%) were sheep. Among these, 25 (46%)—21 goats and 4 sheep—had malformations associated with the consumption of toxic plants. Animals without malformations, those that died due to infectious causes, or those with other causes unrelated to teratogenic plant exposure, such as hereditary diseases, were excluded from this group.

One goat fetus with malformations was also infected with *Leptospira* sp., as identified through PCR. This animal was included in the study because it presented malformations associated with teratogenic plants. In the fetuses with congenital malformations, no histopathological lesions indicative of infections—such as inflammation, hemorrhages, necrosis, or the presence of infectious agents—were observed.

**Figure 4:**
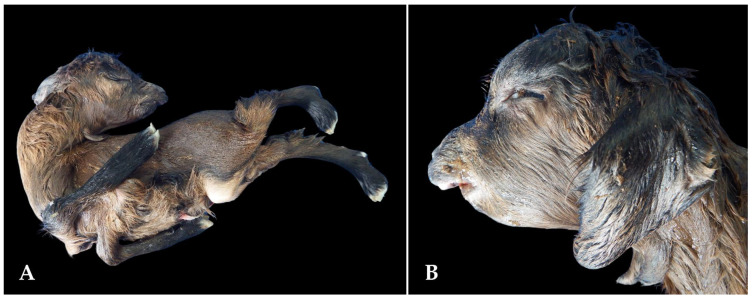
Congenital malformations in goat fetuses associated with the consumption of teratogenic plants in the Brazilian semi-arid region. (**A**) Goat fetus exhibiting arthrogryposis in the thoracic limbs, hyperextension in the pelvic limbs, and brachygnathia. (**B**) Severe fetal brachygnathia.

**Figure 5:**
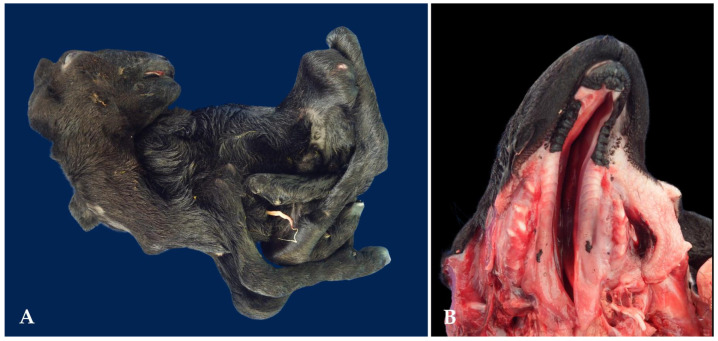
Congenital malformations in stillborn and fetal sheep associated with the consumption of teratogenic plants in the Brazilian semi-arid region. (**A**) Stillborn sheep exhibiting severe arthrogryposis in all four limbs. (**B**) Cleft palate in a sheep fetus.

**Figure 6:**
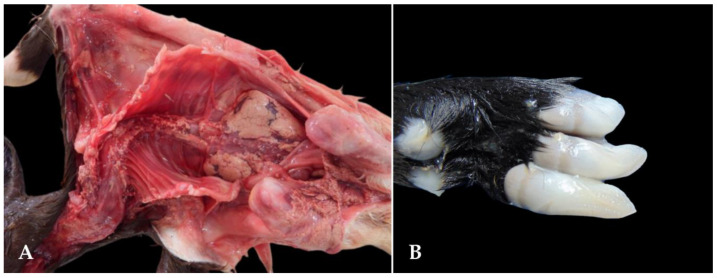
Congenital malformations in goat fetuses associated with the consumption of teratogenic plants in the Brazilian semi-arid region. (**A**) Severe scoliosis. (**B**) Polydactyly.

Similarly to the clinical evaluation of animals on farms, the primaryabnormalities included arthrogryposis and facial malformations (Figure 4 and Figure 5). Other malformations, such as spinal abnormalities and polydactyly (Figure 6), were less common. The animals originated from 21 different farms.

The evaluation of adult females in these herds revealed that 36 females in early pregnancy (up to 45 days) experienced embryonic death and returned to estrus within 25–30 days. Among 20 goats and 1 ewe that were mothers of the fetuses sent for necropsy, dystocia was identified in 6 goats. Of these, four goats died due to birthing complications. Two goats underwent cesarean sections and survived.

The presence of teratogenic toxic plants was identified on farms with cases of congenital malformations. *C. pyramidalis* was recorded on 11 farms (84.62%) and *M. tenuiflora* on 10 farms (76.92%). Both plants was observed on eight farms (61.54%). The types of malformations identified are detailed in Table 3.

Histopathological and molecular evaluations did not identify infectious agents or lesions indicative of infections in fetuses or neonates with congenital defects submitted for necropsy.

### 3.3. Statistical Evaluation

Descriptive analysis revealed that farms using the semi-intensive system had an average of 3.49 ± 5.46 malformations per farm, while the extensive system exhibited an average of 8.05 ± 13.66 malformations. No malformations were reported in the intensive system, making it unfeasible to include this group in subsequent statistical analyses.

The Shapiro–Wilk test indicated that malformation data in the semi-intensive and extensive systems did not follow a normal distribution (*p* < 0.05). The Mann–Whitney U test found no statistically significant differences between the distributions of malformations in the semi-intensive and extensive systems (U = 524.5, *p* = 0.239). Spearman’s correlation revealed a very weak and non-significant positive correlation between the farming system and the number of malformations (R = 0.103, *p* = 0.364).

Among the 42 farms with malformations, farms adopting zootechnical control had an average of 5.55 ± 4.44 malformations (*n* = 11), while farms without zootechnical control recorded an average of 10.16 ± 11.50 malformations (*n* = 31). Point-biserial correlation analysis revealed a correlation coefficient (R) of 0.20, with a *p*-value of 0.205, indicating a weak and non-significant positive correlation between the absence of zootechnical control and increased malformations.

Student’s *t*-test revealed a mean difference of −4.61 malformations between the two groups (t = −1.88; *p* = 0.068). Although the *p*-value for the *t*-test was marginally non-significant, the analysis suggests a trend toward a higher occurrence of malformations on farms without zootechnical control. However, the variability in the group without zootechnical control was substantially higher, as evidenced by the larger standard deviation.

## 4. Discussion

The results of this study demonstrated a high percentage of kids and lambs affected by malformations in the investigated herds, confirming that teratogenic plants are responsible for significant losses in these herds [6,7]. The data on developmental abnormalities in this study are considerably higher than those found in studies from other regions. For example, studies on diseases diagnosed in goats and sheep in Southern Brazil found rates of developmental abnormalities close to 1% [31,32,33,34]. Similarly, an investigation involving more than 600 herds in Iran reported a low prevalence of malformations [35]. On the other hand, research conducted in the semi-arid region of Brazil identified malformation rates of 2.4% in goats and 5% in sheep [13]. It is important to note that the percentage of small ruminants affected by congenital malformations on these farms is likely to be considerably higher, as only live animals with malformations were counted during the first phase of the research. Observational data from farmers regarding malformations in aborted fetuses were not considered due to the probable occurrence of undetected abortions on the farms. This is especially true because most herds were managed under extensive or semi-intensive systems, which made it difficult to observe abortions in pregnant goats and sheep that occurred in open or wooded areas.

The extensive system was the predominant type adopted on the investigated farms. This system has been widely used in the semi-arid region for many years, characterized by low technological input and minimal changes over time [36]. According to Costa et al. [37], in “Cariri Paraibano”, the extensive system accounts for 65% of herds, followed by the semi-intensive system at 33%. The intensive system represents only 2%. The two more commonly adopted systems—extensive and semi-intensive—allow greater access of females to teratogenic plants and limit the ability to control their diets [10]. Additionally, many farms had large areas of native forest containing the two primary teratogenic plants of the Brazilian Northeast [6,7].

Farms using the extensive system demonstrated a higher number of malformations. Although zootechnical control did not show a statistical significance correlation with malformations, properties lacking herd record-keeping systems are less likely to identify and manage pregnant females properly [3]. In extensive systems, animals graze freely and often have prolonged exposure to toxic plants, increasing the likelihood of ingestion [13]. This management approach also hinders the implementation of preventive measures against plant intoxication [10]. Conversely, the intense and prolonged droughts experienced in the region over the last decade [38] led many farms to transition from the extensive system to the semi-intensive system. During drought periods, producers increasingly adopted supplementation through feed troughs, although often in a rudimentary manner [3]. Despite this shift, both systems still allow access to native vegetation [37], enabling the ingestion of teratogenic plants.

The absence of malformations in the intensive farming system can be attributed to the controlled environment, which prevents access to toxic plants. This system restricts animal exposure to native shrubs and trees—plants that are drought-resistant but often toxic—during periods of reduced availability of grasses or annual legumes [9]. Thus, it is evident that management practices play a crucial role in preventing or precipitating livestock poisoning by plants, as identified in other regions [39]. Although this system may not be practical for all phases of small ruminant farming in the semi-arid region, it could be specifically implemented for pregnant females during critical periods of embryonic development. Under this system, animals are not allowed to graze; instead, pregnant goats and sheep are housed and provided with forage, mineral supplements, and concentrated feed. All food is provided in the trough, and their production levels are generally higher than in other systems [4].

Although statistical analyses did reveal a significant negative correlation between the semi-intensive system and malformations, this offers better opportunities for implementing controlled management practices. These practices can include the identification and isolation of pregnant females, reducing their access to toxic plants [3]. However, this system may still be linked to a higher incidence of intoxication in sheep and goats when adopted solely at the end of the dry season and subsequently abandoned after the first rains, particularly on farms with significant infestations of teratogenic plants [9]. The grain-based feed used in semi-intensive systems supports the onset of estrus in females. However, early rainfall during the rainy season is often insufficient to stimulate the growth of most forage plants, while toxic plants may regrow following precipitation events [17]. Consequently, pregnant females may rely on toxic plants as their only source of green forage and fiber at the beginning of gestation [9], increasing the risk of developmental defects in the fetuses.

It has been established that *M. tenuiflora* is the primary cause of malformations in goats and sheep in Northeastern Brazil [19,21]. Although the toxic compounds responsible for the plant’s teratogenic effects have not been fully characterized, strong evidence suggesting a relationship with N-methyl- and N,N-dimethyltryptamine [40]. The specific gestational period during which the plant exerts its teratogenic effects, leading to malformations, is also poorly defined. However, existing evidence indicates that the most susceptible stage is within the first 60 days of gestation [10]. Goats readily consume the sprouts of this plant, which likely explains the higher incidence of malformations in this species, as demonstrated in the present study and previous investigations [7].

*C. pyramidalis* was also associated with malformations in the present study. This association is attributed to the plant’s endemic presence in the study region and its ability to remain viable, retaining a high percentage of leaves even during drought periods [41], making it readily available as feed for the animals. An experimental study on pregnant ewes that received crushed dry leaves of this plant mixed with concentrated feed at concentrations of 20% and 40% resulted in weak premature lambs or aborted malformed fetuses, respectively [42]. Another experiment demonstrated that pregnant goats fed fresh leaves exhibited abortions. Furthermore, goats fed 80% crushed dry leaves aborted after 90 days of gestation, indicating that the plant’s leaves retain their toxicity even after dehydration [16]. A third experiment involving pregnant ewes resulted in newborn lambs displaying several abnormalities similar to those observed in natural cases in our investigation, including brachygnathia, scoliosis, arthrogryposis, rudimentary tongue, slight tortuosity of the proximal phalanges of the thoracic limbs, and tail deviation [15]. Less frequent abnormalities, such as hypoplasia of the lung lobes and polydactyly, were also observed in experimental studies [12,18]. Approximately 20 chemical compounds have been identified in the leaves of *C. pyramidalis*. However, the metabolites responsible for these abnormalities remain undefined [6].

In addition to these two plants, the investigation identified the presence of *A. pyrifolium* on the farms, which may also be associated with embryonic and fetal mortality due to its abortive effect [22]. However, since this plant is not associated with fetal malformations, its effects were not the focus of this study.

Other potential teratogenic agents were considered and differentiated from our cases. For example, changes in serum levels of vitamins and minerals have been associated with congenital defects in the tissues of calves, lambs, and kids, particularly affecting the digestive and urogenital systems [43]. In contrast, malformations caused by teratogenic vegetation predominantly affect the skeletal system or the face, as seen in goats and sheep and described in previous studies [6,7]. Additionally, over 80% of the herds in this study received mineral supplementation, consistent with findings reported by other authors [44].

Elevated levels of mycotoxins in feed can also induce malformations in various organs [26]. However, the occurrence of malformations in this study was lower in semi-intensive herds and absent in confined herds. Inbreeding indices were also low due to investments in genetic improvement programs supported by state government initiatives, such as the “Milk Program of Paraíba” [44]. These efforts have reduced the likelihood of hereditary abnormalities and further reinforced the role of toxic plants in the occurrence of malformations in extensively raised animals in the region.

## 5. Conclusions

This study confirmed a strong association between the consumption of the teratogenic toxic plants *M. tenuiflora* and *C. pyramidalis* and the occurrence of congenital malformations, abortions, and neonatal mortality in goats and sheep raised in northeastern semi-arid regions, resulting in significant economic losses for these farms. Additionally, the absence of histopathological or molecular evidence of infections in animals with congenital defects submitted for necropsy supports the etiology linked to teratogenic plants.

These findings underscore the importance of implementing preventive strategies on farms in the Brazilian semi-arid region. These strategies should focus on identifying and managing grazing areas to minimize pregnant females’ exposure to these toxic plants.

## Figures and Tables

**Table 1:** Characteristics of 80 farms investigated for the occurrence of congenital malformations associated with the ingestion of teratogenic plants by goats and sheep in the Brazilian semi-arid region.

Farm Characteristics	Number	%
Number of Animals on the 80 Farms		
Goats	5988	86.7
Sheep	921	13.3
Type of Production		
Dairy Farms	30	37.5
Meat Farms	26	32.5
Mixed Farms	24	30
Animal Categories		
Adult Females	4241	19.6
Breeding Males	199	2.8
Young Animals *	1352	19.6
Weaned Animals **	1117	16.2

* Suckling animals up to six months old; ** weaned animals in the growth, fattening, or finishing phase.

**Table 2:** Types of congenital malformations identified during the clinical examination of 415 goats and sheep from 42 farms in the Brazilian semi-arid region.

Congenital Malformations	Number of Cases		
	Goats	Sheep	Total
Arthrogryposis	176	17	193
Cleft lip	24	4	28
Cleft palate	4	2	6
Arthrogryposis + Cleft lip + Cleft palate	97	15	112
Arthrogryposis + Cleft lip	41	0	41
Arthrogryposis + Cleft palate	32	0	32
Communicating cleft lip and palate	2	1	3
Total	376	39	415

**Table 3:** Malformations caused by toxic plants in fetuses and stillbirths of goats and sheep from the Brazilian semi-arid region submitted for necropsy.

Diagnostic	Animal Species	Number of Cases *
Arthrogryposis limited to the thoracic limbs	Goat	15
Micrognathia	Goat and Sheep	11
Arthrogryposis in both thoracic and pelvic limbs	Goat and Sheep	10
Inner ear malformations	Goat	7
Hyperextension of limbs	Goat	6
Facial asymmetry malformation	Goat	5
Microphthalmia	Goat	4
Kyphoscoliosis	Goat	4
Cleft palate	Goat and Sheep	3
Nasolabial cleft	Goat and Sheep	3
Scoliosis	Goat	2
Communicating cleft lip and palate	Goat	2
Bilateral polydactyly	Goat	2
Bilateral anophthalmia	Goat	1
Hypoplasia of the lung lobes	Goat	1
Prosencephalic hypoplasia	Goat	1

* Each animal exhibited two or more concomitant malformations.

## Data Availability

The raw data supporting the conclusions of this article will be made available by the authors upon request.
